# Research on WSN reliable ranging and positioning algorithm for forest environment

**DOI:** 10.1038/s41598-024-56180-5

**Published:** 2024-03-05

**Authors:** Peng Wu, Le Yu, Xiaomei Yi, Liang Xu, LiJuan Liu, YuTong Yi, Tengteng Jiang, Chunling Tao

**Affiliations:** 1 College of Mathematics and Computer Science, Zhejiang A & F University, Hangzhou, 311300 People’s Republic of China; 2 College of Humanities and Development Studies, China Agricultural University, Beijing, 100091 People’s Republic of China

**Keywords:** Wireless sensor network (WSN), Ranging positioning, Fitting method, Regional division, High precision, Biogeochemistry, Environmental sciences, Solid Earth sciences

## Abstract

Wireless sensor network (WSN) location is a significant research area. In complex environments like forests, inaccurate signal intensity ranging is a major challenge. To address this issue, this paper presents a reliable WSN distance measurement-positioning algorithm for forest environments. The algorithm divides the positioning area into several sub-regions based on the discrete coefficient of the collected signal strength. Then, using the fitting method based on the signal intensity value of each sub-region, the algorithm derives the reference points of the logarithmic distance path loss model and path loss index. Finally, the algorithm locates target nodes using anchor nodes in different regions. Additionally, to enhance the positioning accuracy, weight values are assigned to the positioning result based on the discrete coefficient of the signal intensity in each sub-region. Experimental results demonstrate that the proposed WSN algorithm has high precision in forest environments.

## Introduction

Positioning technology for wireless sensor networks in forests is critical for ensuring the security of forest resources, social stability, and the safety of people's lives and property^1,2^. Accurately, quickly, and effectively monitoring and detecting abnormal events in forest environments has become a major concern for society. However, the complexity, variability, and danger of the forest environment make it difficult for traditional technologies such as geographic information systems (GIS), remote sensing (RS), and global positioning systems (GPS) to accurately locate and monitor changes in the forest environment^3^.

Wireless sensor networks allow nodes to reach and monitor forest environments that are inaccessible or too dangerous for humans, enabling more precise monitoring and positioning. Nodes in wireless sensor networks are typically classified into anchor nodes (Beacon) or unknown nodes (Unknown)^4–6^, depending on the perspective. Anchor nodes can obtain their location information through manual deployment, while unknown nodes acquire their location information through the node positioning process. Location information is crucial for monitoring and locating activities, and without it, nodes are meaningless.

Wireless sensor network positioning technology can be divided into two types: range-based^7–9^ and range-free^10,11^ positioning methods. Distance-based positioning methods obtain distance or angle information between nodes through technologies such as Received Signal Strength Indicator (RSSI)^12,13^, Time Difference of Arrival (TDOA)^14,15^, and Time of Arrival (TOA)^16,17^ to achieve monitoring and positioning. Distance-independent positioning methods do not require distance or angle information between nodes, but instead use network connectivity and other information to achieve positioning. Common algorithms include Approximate Point-In-Triangulation (APIT)^18–20^, Centroid Method^21^, Distance Vector Hop (DV-Hop)^22–24^, and others.

The RSSI ranging and positioning algorithm is widely used due to its simplicity and lack of additional hardware requirements. Qi et al.^25^ used weighted averages to estimate environmental factor parameters, dynamically modified ranging values, and combined them with the weighted Triangle Centroid Algorithm to achieve positioning. Singh^26^ discussed the challenges and potential solutions related to machine learning-based indoor localization systems. Literature^27^ proposed a new method for evaluating WSN technology for indoor localization using weight range localizer (WRL) and relative span exponential weight range localizer (RS-WRL)^28,29^ based on the RSSI to estimate the target node's position. This algorithm effectively improves positioning accuracy and reduces errors compared to the RSSI algorithm with a fixed loss factor. However, the RSSI value is easily affected by the environment, and the simple loss model's accuracy is not high. Therefore, this paper proposes a WSN reliable location algorithm suitable for forest environments. The logarithmic path loss parameters are determined by the zonal fitting method, and the location is achieved through trilateral ranging. Finally, the RSSI discrete coefficient is used to assign weight values to the location results of each region to improve the positioning accuracy. The signal strength RSSI variations caused by shadow fading for changing AP heights are used to estimate the location accuracy. The localization performance is computed in terms of the Cramer–Rao lower bound (CRLB) of range estimate under dynamic environments^30^.

## The ranging model

Section “Logarithmic distance path loss model” of this chapter mainly introduces the method of calculating the distance based on the signal strength of the logarithmic distance path loss model, while Section “Fitting method to get model parameters” focuses on establishing a suitable logarithmic distance path loss model in a specific environment, such as the forest environment. This section mainly discusses how to fit the path loss index and reference point signal strength of the logarithmic distance path loss model based on measured values.

### Logarithmic distance path loss model

In the forest environment, obstacles are complex and the signal strength (RSSI) is greatly affected. However, the logarithmic distance path loss model fully considers the influence of obstacles and uses the path loss index to represent the complexity of obstacles. Therefore, the logarithmic distance path loss model is widely used to calculate the loss of signal strength RSSI and the corresponding distance in this environment. Equation (1) shows the specific model:

1 $$P\left( d \right) = P_{0} \left( {d_{0} } \right) \pm 10nlg\left( {\frac{d}{{d_{0} }}} \right) + X_{\sigma }$$

In Eq. (1), ${d}_{0}$ is the reference distance, usually 1 m; d is the distance of signal propagation, that is, the distance from the signal source; ${P}_{0}\left({d}_{0}\right)$ is the power path loss at the reference distance ${d}_{0}$; ${P}_{\left(d\right)}$ is the power path loss at the signal propagation distance $d$; $n$ is the path loss index, $n$ represents the rate at which the signal loss changes with the propagation distance, and its value is mainly related to the complexity of environmental obstacles. is the Gaussian error. Convert Eq. (1) to signal strength RSSI as shown in Eq. (2).

2 $$RSSI\left( d \right) = RSSI\left( {d_{0} } \right) - 10nlg\left( {\frac{d}{{d_{0} }}} \right) + X_{\sigma }$$

In Eq. (2), $RSSI\left(d\right)$ is the signal strength path loss value at the distance source $d$; $RSSI\left({d}_{0}\right)$ is the signal strength path loss value from the signal source ${d}_{0}$.

The signal strength at the distance from source d is shown in Eq. (3).

3 $$RSSI_{d} = RSSI_{0} + RSSI\left( d \right)$$

In Eq. (3), ${RSSI}_{d}$ is the signal strength value at the distance source $d$; ${RSSI}_{0}$ is the signal strength value at the transmitting source; $RSSI\left(d\right)$ is the path loss value at the distance source $d$.

The path loss value for the signal strength from the reference point at source ${d}_{0}$ is shown in Eq. (4).

4 $$RSSI\left( {d_{0} } \right) = A - RSSI_{0}$$

In Eq. (4), A is the signal strength value of the reference point at the distance source ${d}_{0}$; $RSSI\left({d}_{0}\right)$ is the signal path loss value at the reference point; ${RSSI}_{0}$ is the signal strength value at the transmitting source.

Substituting Eqs. (3) and (4) into Eq. (2) yields the conversion formula for signal strength to distance, as shown in Eq. (5).

5 $$d = d_{0} \times 10^{{\frac{{A - RSSI_{d} }}{10n}}}$$

In Eq. (5), ${d}_{0}$ is $t$ distance; $A$ is the signal strength value at the reference point; $n$ is the path loss index; In order to calculate the distance based on the signal strength more accurately, ${RSSI}_{d}$ takes the average $\overline{{RSSI }_{d}}$ of the multiple signal strengths at $d$.

### Fitting method to get model parameters

Section “Logarithmic distance path loss model” of this chapter explains how to calculate the distance from a signal source using the signal strength and the logarithmic distance path loss model, which is expressed by Eq. (5). The accuracy of this model depends on the values of the model parameters, including the path loss index ($n$), the reference point (${d}_{0}$), and the signal strength of the reference point ($A$). Thus, this section discusses how to determine these parameters accurately for a specific environment.

**Figure 1:**
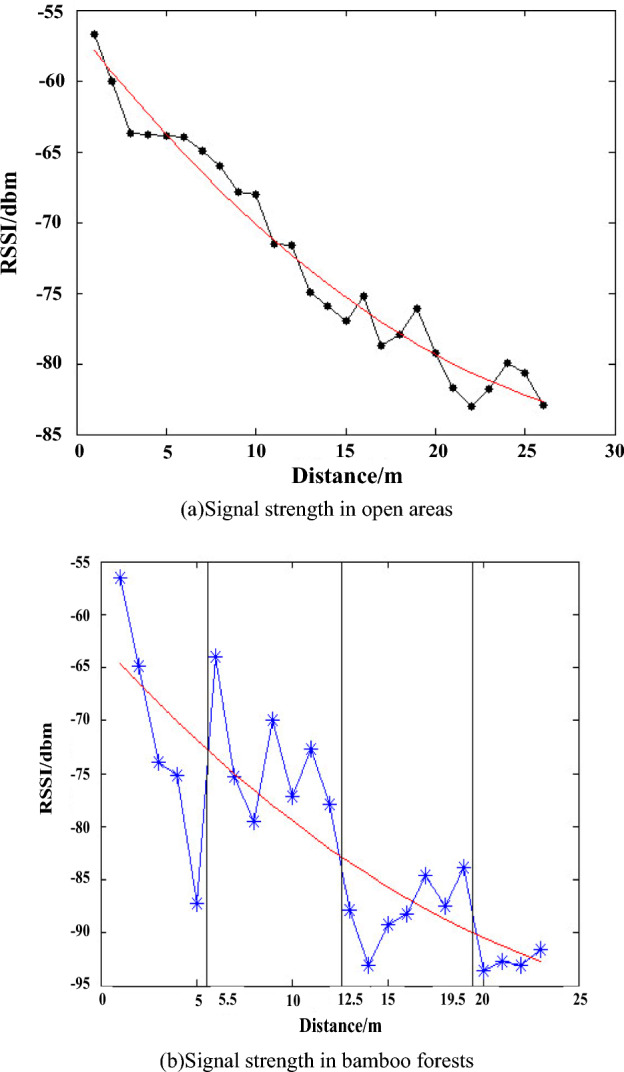
Signal strength measurement results.

In the forest environment, the density of trees varies across different areas, leading to varying signal propagation losses in regions with different densities. Consequently, the path loss index ($n$) and reference point (${d}_{0}$) in the logarithmic distance path loss model also vary. To address this issue, this paper uses the RSSI's discrete coefficient, which varies significantly in regions with different densities, to divide the forest area into regions. To illustrate, the paper presents the actual measured RSSI and distance values in Fig. 1.

In Fig. 1a, the relationship between the signal intensity (*RSSI*) and distance (*d*) is shown for two different environments: an open area and a forest environment (bamboo forest). In open areas, the discrete coefficient of signal strength is relatively stable at around − 0.018. However, in the forest environment, the signal intensity (*RSSI*) is more dispersed, and there are significant fluctuations in RSSI values at 5.5 m, 12.5 m, and 19.5 m from the signal source. These fluctuations are caused by the varying density of trees in different regions. By using the dispersion coefficient of RSSI, the positioning area can be effectively divided, and the path loss index (*n*) and reference point (${d}_{0}$) of the logarithmic distance path loss model can be calculated for each region.

To calculate the path loss index (*n*) and reference point (${d}_{0}$) for each region, a fitting method is used. The *RSSI* values measured in each region are used as the ordinate and distance (*d*) is used as the abscissa in the fitting process. Equation (5) is used as the custom fitting function, and the n linfit nonlinear fitting function of MATLAB is used to fit the parameters ${d}_{0}$, A, and n in Eq. (5). The localization region is divided into four sub-regions, and the model parameters corresponding to each sub-region are calculated as follows: *d*_*01*_, *A*_*1*_, and *n*_*1*_ for sub-region 1, *d*_*02*_, *A*_*2*_, and *n*_*2*_ for sub-region 2, *d*_*03*_, *A*_*3*_, and *n*_*3*_ for sub-region 3, and *d*_*04*_, *A*_*4*_, and *n*_*4*_ for sub-region 4. The logarithmic distance path loss model obtained by fitting can measure the distance more accurately, leading to improved positioning accuracy in ranging and positioning applications.

## Positioning method

Section “Partition positioning” of this chapter focuses on the collaborative localization of unknown nodes using anchor nodes in each region through weight-based triangulation. The goal is to improve the accuracy of localization. In this method, anchor nodes with known locations are used to form a reference network. Each unknown node measures its distance to the anchor nodes using the received signal strength indicator (RSSI) and then sends these measurements to a central processor. The central processor then calculates the location of the unknown node using the weight-based triangulation method. This method takes into account the distances and the corresponding weights assigned to each anchor node, and calculates the weighted average of their locations to obtain the location of the unknown node. The RSS signal strength measurements are made in LOS and OLOS regions to construct and validate the models. The shadow fading component for quasi-realistic and realistic conditions is statistically modeled with the dependency on AP heights^31^.

Section “Fitting method to get model parameters” explains how the discrete coefficient of signal strength is used to assign weight values to each sub-region positioning result, in order to further improve the accuracy of positioning. The weight value is calculated by taking into account the dispersion coefficient of the RSSI in each sub-region. This means that regions with more dispersed RSSI values have lower weight values assigned to their positioning results, and vice versa. By using this method, the positioning accuracy is improved, especially in areas with complex environments such as forests, where the RSSI values are greatly affected by the obstacles.

### Partition positioning

**Figure 2:**
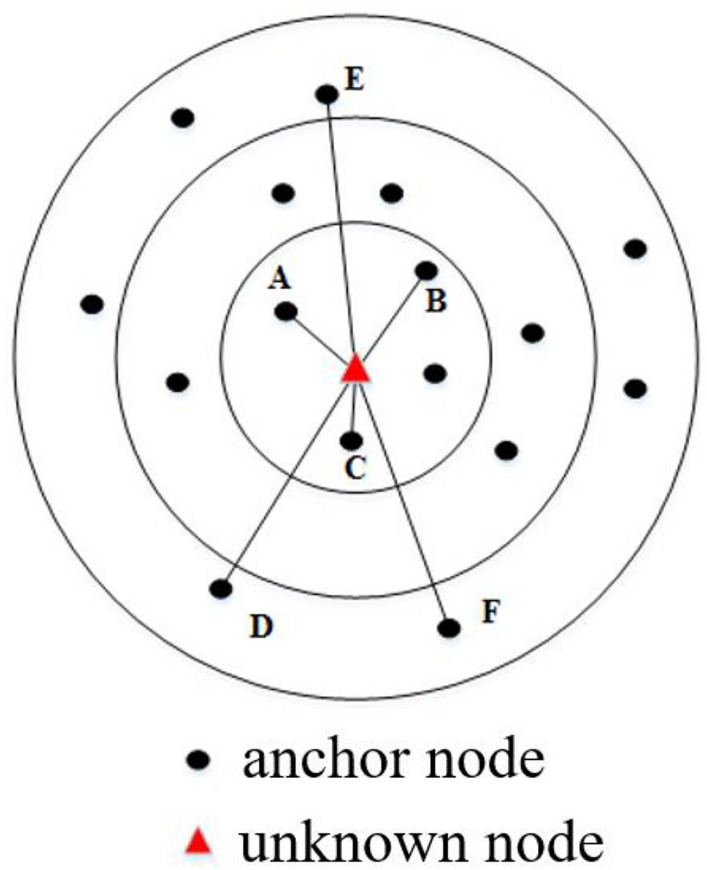
Partition location map.

To improve the positioning accuracy, this paper divides the positioning area into sub-regions based on the discrete coefficient of RSSI, as shown in Fig. 2. Each sub-region is equipped with several anchor nodes. A multi-area anchor node collaborative positioning method is adopted, where the unknown node is located using the anchor nodes in its closest sub-regions. The triangles represent unknown nodes, and the circles represent anchor nodes.

The initial stage involves the selection of anchor nodes proximate to the unknown node within each sub-region for precise positioning. The criteria for selecting these anchor nodes are based on both proximity and signal strength. Specifically, the distance between the unknown node and potential anchor nodes is determined using Received Signal Strength Indicator (RSSI). The anchor nodes with the strongest and most reliable RSSI values in each sub-region are chosen as the anchors for subsequent triangulation ranging.

To illustrate, in area 1, anchor nodes A, B, and C are selected based on their close proximity to the unknown node and their superior RSSI values. Similarly, in area 3, anchor nodes D, E, and F are chosen following the same criteria. This rigorous selection process ensures that the selected anchor nodes are not only geographically close but also exhibit robust signal strength, contributing to the accuracy of the triangulation ranging process. In our approach, we utilize a discrete coefficient derived from the collected signal strength data to delineate sub-regions. The discrete coefficient acts as a measure of signal variability within the environment, aiding in the identification of areas with distinct signal characteristics. The algorithm dynamically adapts to variations in signal strength by adjusting the boundaries of these sub-regions.

Taking region 1 as an example, assuming the coordinates of the unknown node are *(x*_*1*_*, y*_*1*_*)*, the coordinates of anchor nodes A, B, and C in area 1 are* (x*_*a*_*, y*_*a*_*)*,* (x*_*b*_*, y*_*b*_*)*, and *(x*_*c*_*, y*_*c*_*)*, and the signal strength reaching the unknown node is *RSSI*_*A*_, *RSSI*_*B*_, and *RSSI*_*C*_. The distance between the anchor node and the unknown node can be calculated using the signal strength, i.e., *d*_*a*_, *d*_*b*_, and *d*_*c*_. Finally, the coordinates of the unknown node can be obtained using formula (6).

6 $$\left\{ {\begin{array}{*{20}l} {\left( {x_{1} - x_{a} } \right)^{2} + \left( {y_{1} - y_{a} } \right)^{2} = d_{a}^{2} } \hfill \\ {\left( {x_{1} - x_{b} } \right)^{2} + \left( {y_{1} - y_{b} } \right)^{2} = d_{b}^{2} } \hfill \\ {\left( {x_{1} - x_{c} } \right)^{2} + \left( {y_{1} - y_{c} } \right)^{2} = d_{c}^{2} } \hfill \\ \end{array} } \right.$$

### Improve positioning accuracy

Although the logarithmic distance path loss model proposed in this paper can measure ranging more accurately, there are still errors, especially in regions with large RSSI discrete coefficients. In these regions, the RSSI values are unstable and tend to lead to large ranging errors. To improve the positioning accuracy, this paper proposes using the RSSI discrete coefficient to assign weight values to the positioning results of each region.

Assuming that the localization area is divided into s sub-regions with discrete coefficients of signal strength RSSI denoted as *D*_*1*_*, D*_*2,*_* …, D*_*i*_*, …,* and *D*_*s*_ for each region, the larger the dispersion coefficient, the less reliable the positioning results of the anchor nodes in that region are to unknown nodes. On the other hand, the smaller the dispersion coefficient, the more confident the anchor nodes in that region are in the positioning results of the unknown nodes. The weight assignment formula is shown in Eq. (7).

7 $$W_{i} = \frac{1}{{D_{i} \times \mathop \sum \nolimits_{i = 1}^{s} \frac{1}{{D_{i} }}}}$$

Assuming that the anchor node of sub-region *i* locates the unknown node with coordinates *(x*_*i*_*, y*_*i*_*)*, $$i \in (1, s)$$, then the cooperative positioning result of all sub-regions to unknown nodes is obtained by using a weighted average based on the weight values assigned to each sub-region. The coordinates ($$x, y)$$ of the collaborative positioning result can be calculated using Eq. (8): The weight-based multi-region collaborative positioning method proposed in this paper can effectively reduce positioning errors and improve positioning accuracy. By considering the signal strength dispersion of each sub-region, the method assigns appropriate weights to the positioning results of anchor nodes in each sub-region, which can effectively eliminate the influence of unstable RSSI and improve the accuracy of positioning results.

8 $$\left\{ {\begin{array}{*{20}l} {X = \mathop \sum \limits_{i = 1}^{s} \left( {W_{i} \times X_{i} } \right)} \hfill \\ {Y = \mathop \sum \limits_{i = 1}^{s} \left( {W_{i} \times Y_{i} } \right)} \hfill \\ \end{array} } \right.$$

## Experiments and analysis

**Figure 3:**
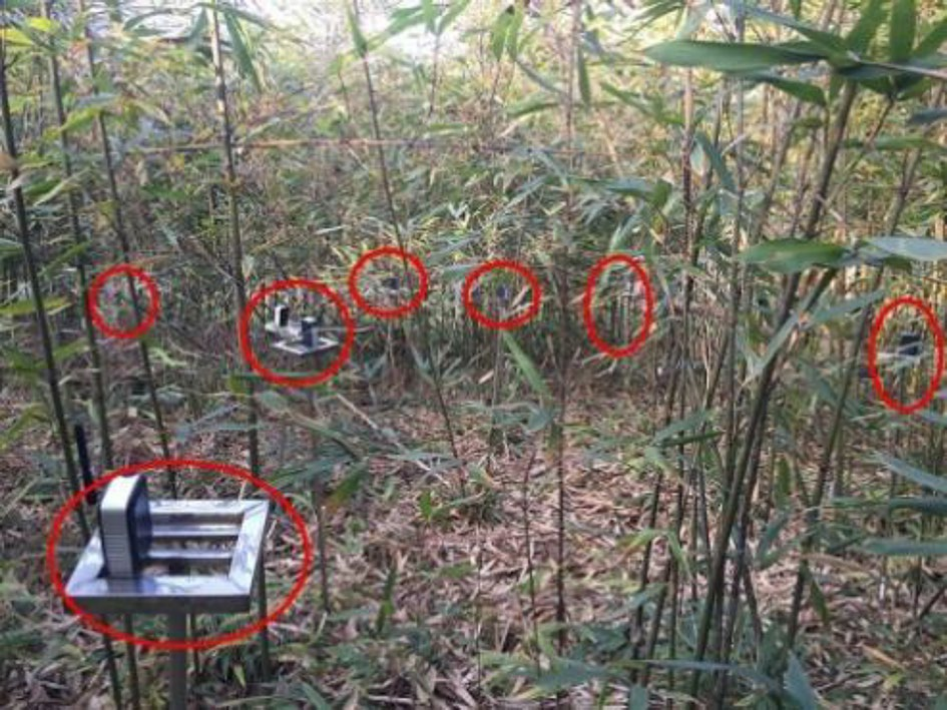
Experimental scene.

In the experiment, a sensor network was established using Telosb sensor nodes. The sensor nodes were evenly deployed in a bamboo forest with a radius of 20 m. The density of the bamboo forest was not uniform, and there were a total of 20 anchor nodes and 10 unknown nodes. The sensor node was installed on a 1.2 m high bracket. During the experiment, the sensor network transmitted the data required for positioning to a PC, which then performed the positioning calculations. Figure 3 shows the experimental setup.

### Distance measurement

**Figure 4:**
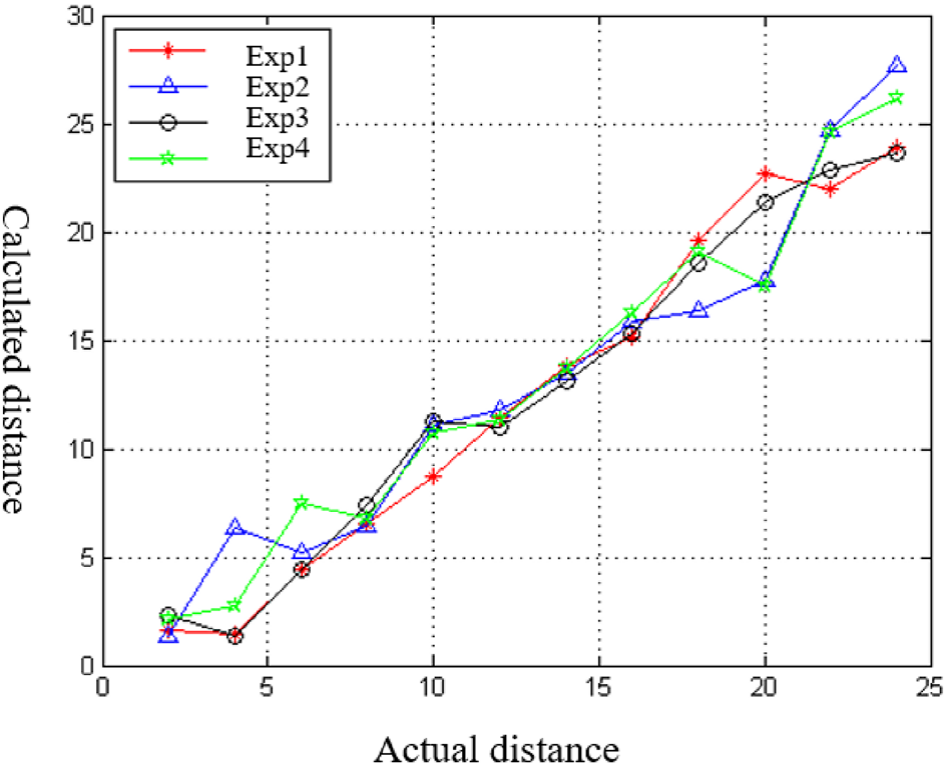
Ranging experiment diagram.

This experiment aimed to verify the accuracy of the proposed ranging method. A signal transmitting node was deployed in the bamboo forest, and a signal receiving node was placed at multiple locations to measure the signal strength (RSSI) and the distance between the nodes. The 1.2 subsection method was used to obtain the parameters *(d*_*0*_*, A, n)* in the logarithmic distance path loss model, which was then used to construct a path loss model specific to the environment. The ranging experiment was carried out using the established model, and the results are presented in Fig. 4. The x-axis represents the actual distance between the receiving node and the transmitting node, while the y-axis represents the distance between the receiving node and the transmitting node calculated by the logarithmic distance path loss model proposed in this paper. The results indicate that the proposed ranging method can accurately measure the distance between nodes in the bamboo forest.

In this experiment, ranging was carried out in four different directions from the transmitting node. The results showed that the proposed method of obtaining the parameters for the ranging model can construct the model more accurately, leading to more accurate ranging in specific environments. The ranging error for the model was found to be approximately 5.3%, which meets the positioning requirements in the complex forest environment. The experiment successfully verifies the effectiveness and accuracy of the ranging method proposed in this paper.

### Multi region collaborative positioning experiment

In this experiment, the aim was to compare the positioning accuracy of regional cooperative positioning and non-regional positioning. The positioning area was divided into four sub-regions using the 2.1 subsection method. The path loss index n for each sub-region was 2.95, 1.63, 2.41, and 1.87, and the reference distance d0 between the reference point and the transmitting node was 1.53 m, 2.17 m, 1.84 m, and 2.06 m, respectively. The signal intensity values A at the reference point positions were − 48.67, − 55.14, − 50.32, and − 53.46, respectively.

For the non-regional positioning method, the path loss index n was 2.48, d0 was 2.23 m, and A was − 56.85, obtained by fitting the model without considering the sub-regions, and the unknown node was located by randomly selecting three anchor nodes.

The weight-based positioning optimization results (“partition positioning-weight”) and positioning average results (“partition positioning-average”) under the situation of regional cooperative positioning were also compared.

**Figure 5:**
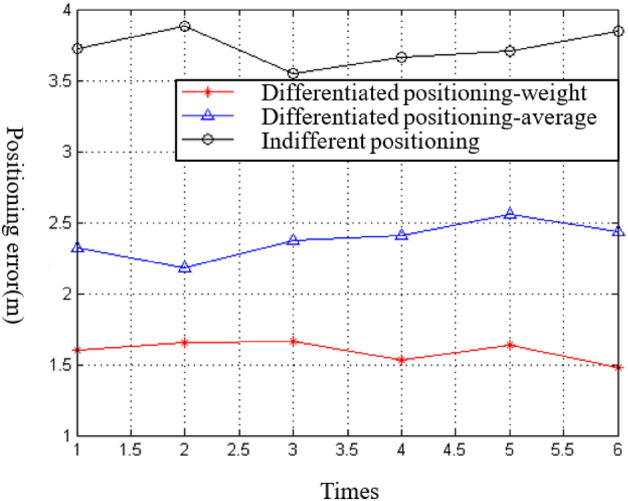
Positioning error diagram.

The experimental results, which are the average positioning error of 10 unknown nodes, are shown in Fig. 5. The abscissa represents the number of experiments, and the ordinate represents the average positioning error.

In this experiment, a comparison was made between regional cooperative positioning and non-regional positioning. The positioning area was divided into sub-regions using the 2.1 subsection method. The path loss index (n) of each sub-region was 2.95, 1.63, 2.41, and 1.87, and the distance (d0) between the reference point and the transmitting node was 1.53 m, 2.17 m, 1.84 m, and 2.06 m, respectively. In addition, the signal intensity values (A) at the reference point positions were − 48.67, − 55.14, − 50.32, and − 53.46, respectively. The path loss index (n) was 2.48, d0 was 2.23 m, and A was − 56.85 by fitting the zonal positioning method, and the unknown node was located by randomly selecting three anchor nodes (“non-zonal positioning”).

The weight-based multi-region collaborative positioning method (“partition positioning-weight”) and the positioning average method (“partition positioning-average”) were compared to non-zonal positioning. The experimental results, which represent the average positioning error of 10 unknown nodes, are shown in Fig. 5. The abscissa represents the number of experiments, and the ordinate represents the average positioning error.

The “partition positioning-weight” method proposed in this paper had the smallest positioning error for 10 unknown nodes, with an error of about 1.55 m. The positioning error of “partition positioning-average” was about 2.4 m, but the error of “non-zonal positioning” was large, about 3.7 m. The logarithmic distance path loss model constructed using the “partition positioning-weight” and “partition positioning-average” methods was more suitable for the positioning environment, and the ranging results were more accurate, resulting in a smaller positioning error compared to “non-zonal positioning”.

Furthermore, the “partition positioning-weight” method optimized the positioning results based on the weight of the signal strength discrete coefficient, making the distance measurement using signal strength more stable. Therefore, the positioning error was smaller than that of the “partition positioning-average” method.

### Comparison of positioning methods

In this study, two ranging models were compared to investigate their impact on the accuracy of the proposed positioning algorithm. The first model was constructed using a fitting method, which is more accurate and better suited to the actual environment than the traditional model that selects a reference point 1 m away from the signal source and uses empirical values for the path loss index. Literature^32^ propose the algorithm that adapts the area location estimation by considering both the packet lost rate and the received signal strength. This adaptive approach aims to enhance the accuracy and reliability of the location estimation in wireless sensor networks. The experiment evaluated the difference in positioning accuracy between the two ranging models, as well as compared the proposed R&PLR-LA positioning algorithm to the one proposed.

**Figure 6:**
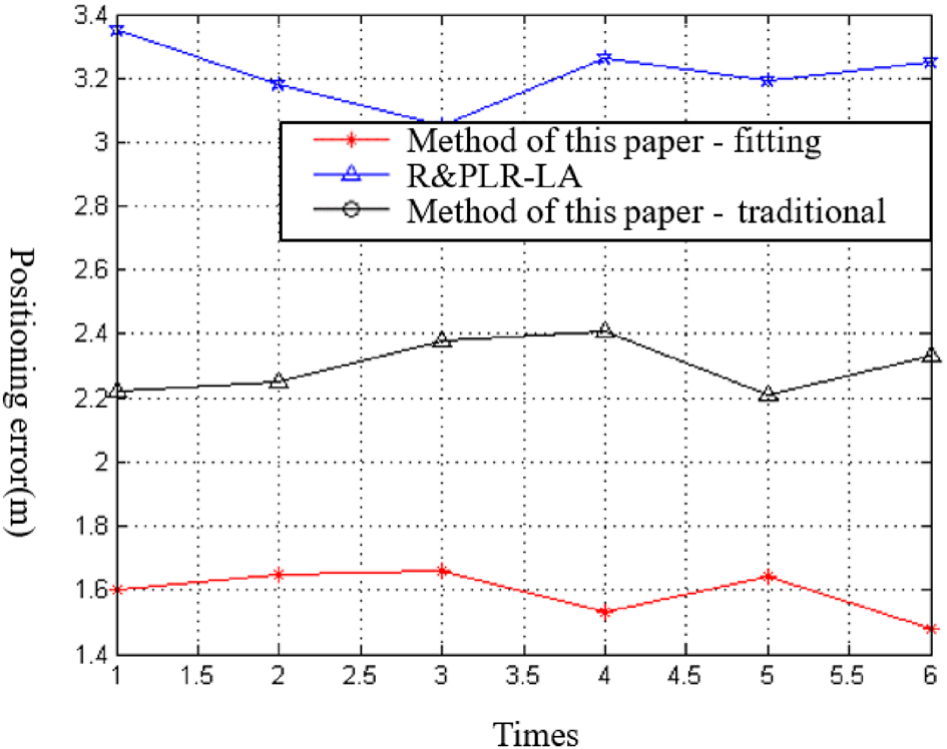
Positioning diagram of the ranging model.

The results show that the ranging model constructed using the fitting method has a smaller positioning error, with an average error of about 1.76 m (“method in this paper—fitting”), compared to the traditional model with an average error of about 2.86 m (“method in this paper—traditional”). Additionally, the R&PLR-LA positioning algorithm proposed in this study has a smaller positioning error compared to the algorithm proposed in literature^32^. The average positioning error of the R&PLR-LA algorithm is about 1.76 m, while the average error of the algorithm proposed in literature^32^ is about 2.41 m. These results demonstrate the effectiveness of the proposed ranging model and the R&PLR-LA algorithm in improving the accuracy of positioning in complex environments. The experimental results are summarized in Fig. 6, where the abscissa represents the number of experiments, and the ordinate represents the positioning error.

The results of the experiment show that the proposed positioning method using the fitting method (“Method—Fitting in this paper”) has a positioning error of about 1.6 m, while the positioning error of the positioning method combining the traditional logarithmic distance path loss model is about 3.2 m. In comparison, the R&PLR-LA positioning method proposed in literature ^14^ has a positioning error of about 2.3 m. Since the path loss model constructed by “Method—Fitting in this paper” conforms more accurately to the specific environment, the ranging results are more accurate, resulting in a lower positioning error than that of “Method in this paper—Traditional”. On the other hand, the R&PLR-LA positioning method does not differentiate between the positioning areas, resulting in a higher localization error compared to “Method—Fitting in this paper”. The results indicate that the proposed method can achieve higher positioning accuracy in complex environments. The experimental results are shown in Fig. 6, where the abscissa represents the number of experiments, and the ordinate represents the positioning error.

## Conclusion

In summary, this paper introduces a robust Wireless Sensor Network (WSN) ranging and positioning algorithm tailored for forest environments. Initially, the algorithm strategically divides the positioning area into sub-regions based on the collected signal intensity values. Subsequently, employing a fitting method, it establishes a logarithmic distance path loss model for each sub-region. Crucially, to optimize the positioning accuracy, the algorithm assigns weight values to the results of each region using the signal intensity discrete coefficient.The e﻿xperimental findings affirm the efficacy of the proposed positioning method in accurately locating nodes within forest environments characterized by obstacles. Notably, our ongoing research will focus on refining the ranging model, aiming to quantifiably enhance the accuracy of signal strength-based ranging in challenging forest conditions. By providing a more quantitative assessment of the improvement in ranging accuracy, we strive to contribute further to the advancement of WSN applications in complex environmental settings.

## Data Availability

The data that support the findings of this study are available from the corresponding author, Xiaomei Yi, upon reasonable request.
